# LAIT: a local ancestry inference toolkit

**DOI:** 10.1186/s12863-017-0546-y

**Published:** 2017-09-06

**Authors:** Daniel Hui, Zhou Fang, Jerome Lin, Qing Duan, Yun Li, Ming Hu, Wei Chen

**Affiliations:** 1 Department of Computer Science, University of Pittsburgh, Pittsburgh, PA 15213 USA; 2 Department of Biostatistics, University of Pittsburgh, Pittsburgh, PA 15213 USA; 3 Department of Human Genetics, University of Pittsburgh, Pittsburgh, PA 15213 USA; 4 Department of Genetics, Curriculum in Bioinformatics and Computational Biology, Department of Statistics, Department of Computer Science, University of North Carolina, Chapel Hill, NC 27599 USA; 5 Department of Biostatistics, Department of Genetics, Department of Computer Science, University of North Carolina, Chapel Hill, NC 27599 USA; 6 Department of Quantitative Health Sciences, Lerner Research Institute, Cleveland Clinic, Cleveland, OH 44195 USA; 7 Division of Pulmonary Medicine, Allergy and Immunology, Department of Pediatrics, Children’s Hospital of Pittsburgh of UPMC, Pittsburgh, PA 15213 USA

**Keywords:** Admixture, Local ancestry inference

## Abstract

**Background:**

Inferring local ancestry in individuals of mixed ancestry has many applications, most notably in identifying disease-susceptible loci that vary among different ethnic groups. Many software packages are available for inferring local ancestry in admixed individuals. However, most of these existing software packages require specific formatted input files and generate output files in various types, yielding practical inconvenience.

**Results:**

We developed a tool set, Local Ancestry Inference Toolkit (LAIT), which can convert standardized files into software-specific input file formats as well as standardize and summarize inference results for four popular local ancestry inference software: HAPMIX, LAMP, LAMP-LD, and ELAI. We tested LAIT using both simulated and real data sets and demonstrated that LAIT provides convenience to run multiple local ancestry inference software. In addition, we evaluated the performance of local ancestry software among different supported software packages, mainly focusing on inference accuracy and computational resources used.

**Conclusion:**

We provided a toolkit to facilitate the use of local ancestry inference software, especially for users with limited bioinformatics background.

**Electronic supplementary material:**

The online version of this article (doi:10.1186/s12863-017-0546-y) contains supplementary material, which is available to authorized users.

## Background

Genetic studies of admixed populations such as Latinos and African Americans have been successful in identifying disease-susceptible loci, which can be difficult to detect by other methods such as genome-wide association studies (GWAS). To perform such analyses, one needs to infer the ancestry origins of two copies of an autosomal allele for each individual at each genetic locus (local ancestry inference). A variety of methods have been proposed to do this analysis effectively and efficiently [1–5].

Many genetic analyses require specific input formats. For example, FASTQ and SAM formats are commonly used for sequencing analysis and PLINK format is used for GWAS. However, this standardization has not been the case in local ancestry analysis, because each software package requires a unique format of input files. In addition, the input files usually require certain pre-processing, such as excluding loci which are not in the subset shared by all files [1, 4], removing all duplicate loci [2, 4], removing all monomorphic heterozygous loci [2], etc. Without such proper data pre-processing, some of the programs will fail, or worse, seemingly work correctly but yield incorrect output. Preparing each individual input file can be labor intensive, especially for users with limited scripting knowledge. Motivated by a widely used file formatting tool Mega2 [6], our toolkit LAIT automatically performs all pre-processing and produces correct formatting from standard PLINK files. Our tool will likely increase the usability of the supported software for users with limited bioinformatics background.

Although a descriptive comparison between different local ancestry software was presented recently [5], quantitative comparisons between various local ancestry inference software need to be further investigated. With the implementation of four commonly-used software packages, LAIT allows users to perform comprehensive comparisons among all supported inference software package. As a pilot study, we compared the performance of all implemented software, mostly in terms of inference accuracy, as well as runtime and computational resources.

### Implementation

**Fig. 1:**
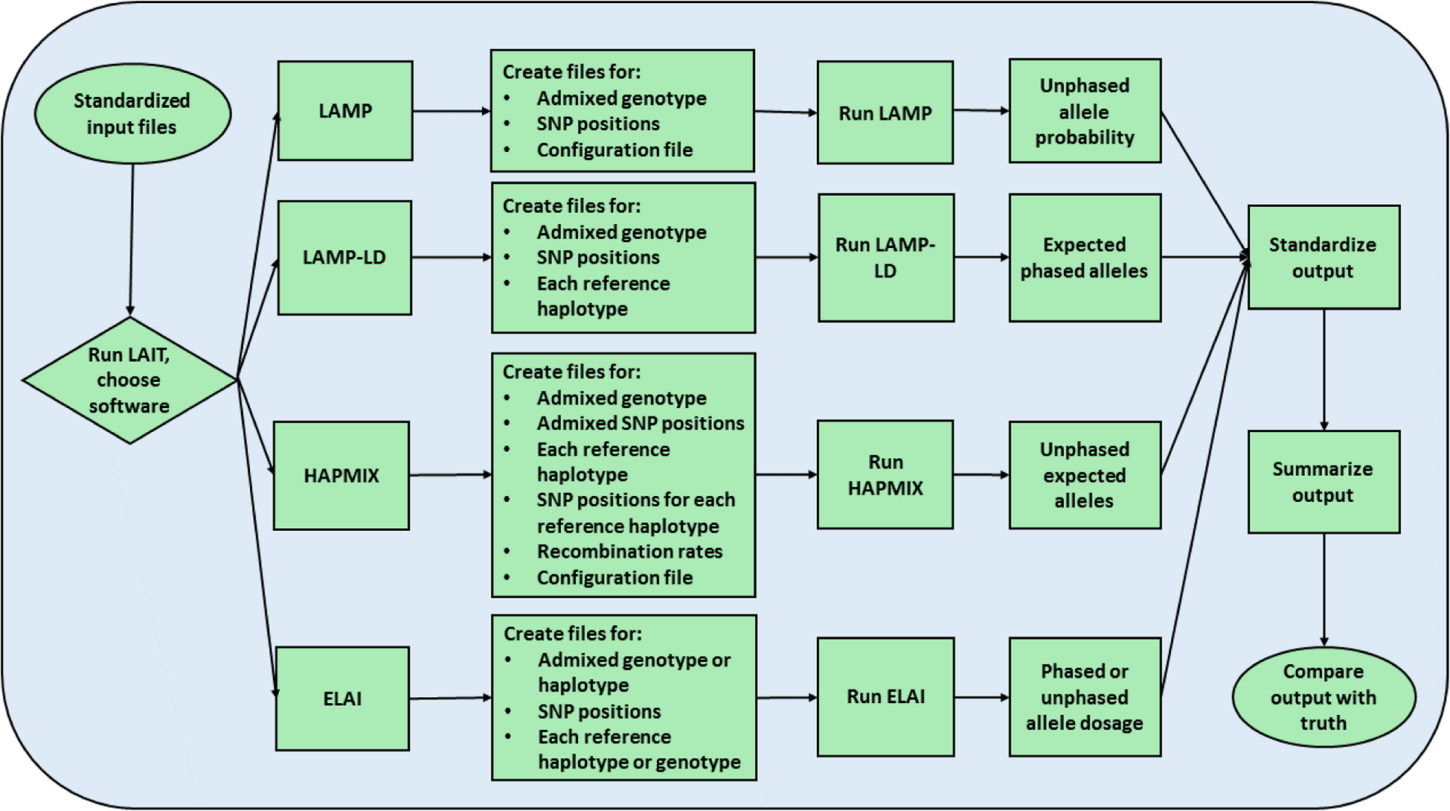
LAIT workflow

We described the workflow in Fig. 1. To reach a broad audience, based on our general knowledge of the field, we have implemented four popular ancestry software packages, including HAPMIX [1], LAMP [2], LAMP-LD [3], and ELAI [4]. Other software can be incorporated into our framework, if needed, in future work.

For the processing of input files, there are multiple parameters for deciding what type of analysis that users want to perform. Each option has different required files, which usually include at least PLINK pedigree and map files, and reference haplotypes or genotypes. LAIT will perform required pre-processing and formatting, which varies for each software but usually consists of changing the coding of the alleles to 0, 1, or 2 corresponding to the number of reference alleles at that locus, removing duplicate sites, only keeping sites that are a subset of all input files, and proper formatting. The output files of the pre-processing step can be used to run each inference software successfully.

**Fig. 2:**
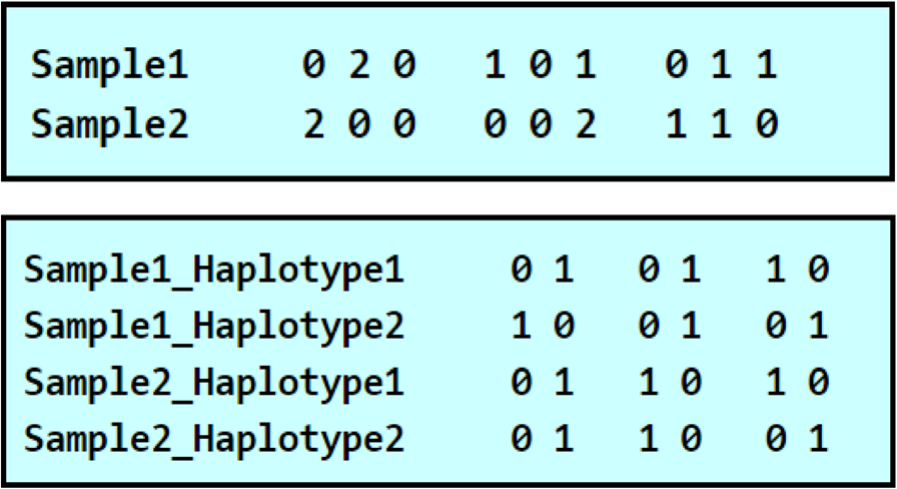
Standardized output format for admixed samples from LAIT. The first diagram is for two samples that are unphased and 3-way admixed. Sample1’s first marker has zero alleles from population one, two alleles from population two, and zero alleles from population three. The second diagram is for two samples that are phased and 2-way admixed. At the first marker Sample1’s first haplotype has zero alleles from population one and one allele from population two

After running an inference software, LAIT has functionality to convert each of the software’s outputs into a standardized form and compute the average ancestry of each. The format, as described in Fig. 2, has one column per marker, with each column denoting how many alleles come from each reference population. If the data is unphased then there is one sample per line, and if the data is phased then there is one haplotype per line (two lines per sample).

### Evaluation of different software

**Fig. 3:**
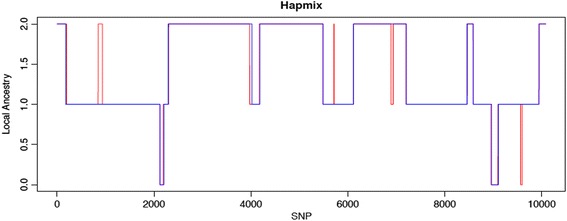
A comparison of true and inferred ancestry using HAPMIX for two-way admixed populations. The y-axis shows how many alleles from the first reference population belong to the SNP at that location. The blue line is the inferred ancestry, and the red is the truth

After the completion of LAIT, we were able to compare the performance of different inference software. Although a comprehensive analysis is beyond the scope of this paper, we tested LAIT on both simulated studies and GWAS data to demonstrate the functionality of LAIT. Different sets of simulated data were created to test all software across inference for two-way and, if supported, three-way admixture. The genetic distance, number of samples, and number of single nucleotide polymorphisms (SNPs) were kept constant across runs for two-way and three-way admixture, for consistency between the comparisons. In order to create the input files, we used an in-house program named SimAdmix, which simulated genetic data from admixed populations using reference data downloaded from The International HapMap Project [7] or 1000 Genomes Project [8]. By comparing the inferred ancestry and true ancestry, the differences in error between all software could be computed – information about the computational resources used was also recorded and compared. As illustrated in Figure 3, the difference between inferred and true ancestry can be visualized in an example run that used HAPMIX. In addition, to demonstrate usability outside of simulated data, we tested the software in a real study on African-American populations. Detailed simulation information can be found in online Additional file 1.

Table 1 shows the inferred ancestries of simulated two-way and three-way admixed samples, as well as the average runtimes and memory usages of all inference software across all samples. For two-way inference, there was a high agreement across all the software. LAMP had the lowest accuracy out of all inference software but also had the lowest resource usage, which was expected as it relies on allele frequencies for inference and does not take advantage of (or requires as input) linkage disequilibrium data, which may be much more informative. For the remaining software, even though they all use linkage disequilibrium data and rely on hidden Markov models as part of their underlying algorithms, HAPMIX had noticeably higher accuracy than the others, but also had much higher resource consumption. For three-way inference (which HAPMIX is not compared due to its limitation), the results were as expected, with LAMP-LD and ELAI having reasonable performance rivalling that of LAMP’S. Overall, accuracy decreased and resource usage increased across all software because multi-way admixture is a more difficult problem due to the greater number of ancestries.

**Table 1:** Simulation results of local ancestry

Ways admixed	Criteria	LAMP	LAMP-LD	ELAI	HAPMIX
Two-way	Mean Squared Error	.399	.156	.144	.004
	Alleles Correct (%)	83.1	92.2	93.6	99.8
	Runtime (minutes)	.497	8.86	10.6	42.9
	Max Memory Usage (GB)	.103	.217	.090	1.01
Three-way	Mean Squared Error	1.08	.305	.503	-- ^a^
	Alleles Correct (%)	62.9	84.9	82.7	-- ^a^
	Runtime (minutes)	.414	14.7	25.4	-- ^a^
	Max Memory Usage (GB)	2.69	.287	.137	-- ^a^

^a^HAPMIX can only do 2-way inference

In addition to simulated data, we applied LAIT to a cohort of African Americans, which have whole-genome SNP data. Since there was no truth to compare with the inferred outputs, we averaged the ancestry between all samples and all chromosomes and compared it to the expected ancestry from other studies [9, 10]. We observed high consistency (Table 2) among all software packages and strong similarity to the cited studies.

**Table 2:** Average global proportion of inferred African ancestry in African Americans

Reference	LAMP	LAMP-LD	ELAI	HAPMIX
CEU	.29	.24	.24	.28
YRI	.71	.76	.76	.72

Furthermore, we calculated the correlation between local inference results between all supported software, in order to inspect the local inference opposed to only the global. From the results in Table 3, it can be seen that the local inference results in real data strongly relate to the results from the simulated data. As LAMP had the lowest accuracy of the supported software, its result also had the lowest correlations between the others – meanwhile, HAPMIX’s results also did not have much larger correlation between ELAI’s and LAMP-LD’s, given that it’s two-way inference was more accurate. As expected, LAMP-LD and ELAI’s results had the highest correlation between each other, as they also had the most similar inference results on the simulated data.

**Table 3:** Average Pearson correlation (r) between local ancestry inference in African Americans

	LAMP-LD	HAPMIX	ELAI
LAMP	.66	.60	.70
LAMP-LD	--	.73	.89
HAPMIX	--	--	.75

## Results and Discussion

We will pursue several future directions to extend LAIT. One is to add more inference software to LAIT. We will focus on software that are following in popularity from the ones already supported, or others that are new and boast enhanced performance (e.g. RFMix [11]). Furthermore, we will perform a more comprehensive comparison on the supported software to examine the impact of the track length, the number of generations, the number of SNPs and individuals on local ancestry inference.

## Conclusions

We present a toolkit that is capable of conveniently pre-processing, cleaning, and formatting from standardized inputs for a variety of popular local ancestry inference software, as well as having additional functionality to standardize and summarize output. Additionally, we present results for a basic comparison between all supported inference software, highlighting various pros and cons of each.

## Availability and requirements

**Data availability:** The datasets used in the current study are available from the corresponding author on request.

**Project name:** Local Ancestry Inference Toolkit.

**Project home page:** http://www.pitt.edu/~wec47/lait.html

**Program used for simulation study:** http://www.pitt.edu/~wec47/simadmix.html

**Operating system(s):** Platform independent.

**Programming language(s):** (1) Perl, (2) C++.

**Other requirements:** No.

**License:** *GNU General Public License *(June 29, 2007). Version 3. Free Software Foundation. URL: https://www.gnu.org/licenses/gpl-3.0.en.html.

**Any restrictions to use by non-academics:** No.

## Additional file

Additional file 1: Simulation details of comparison analysis. (DOCX 14 kb)

## Abbreviations

GWAS: Genome-wide association study
LAIT: Local Ancestry Inference Toolkit
SNP: Single nucleotide polymorphism
