# Supplementary material for: LAIT: a local ancestry inference toolkit
Source: BMC Genet. 2017 Sep 6;18:83. doi: 10.1186/s12863-017-0546-y (PMC5585928; doi:10.1186/s12863-017-0546-y)
Supplement: Additional file 1: — Simulation details of comparison analysis. (DOCX 14 kb) [file 12863_2017_546_MOESM1_ESM.docx]

LAIT: a local ancestry inference toolkit

Daniel Hui^1^, Zhou Fang^2^, Jerome Lin^3^, Qing Duan^4^, Yun Li^5^, Ming Hu^6^, and Wei Chen^2,3,7*^

^1^Department of Computer Science, ^2^Department of Biostatistics, ^3^Department of Human Genetics, University of Pittsburgh, Pittsburgh PA, 15213.

^4^Department of Genetics, Curriculum in Bioinformatics and Computational Biology, Department of Statistics,^5^Department of Biostatistics, Department of Genetics, Department of Computer Science, University of North Carolina, Chapel Hill, NC 27599, USA.

^6^Department of Quantitative Health Sciences, Lerner Research Institute, Cleveland Clinic, Cleveland OH, 44195.

^7^Division of Pulmonary Medicine, Allergy and Immunology, Department of Pediatrics, Children’s Hospital of Pittsburgh of UPMC, Pittsburgh PA, 15213

^*^ Correspondence: Wei Chen [wei.chen@chp.edu](mailto:wei.chen@chp.edu)

daniel.hui@pitt.edu^1^, fangz.ark@gmail.com^2^, emilio0924@gmail.com^3^, qduan@email.unc.edu^4^, yunpersonal@gmail.com^5^, afhuming@gmail.com^6^, wei.chen@chp.edu^7^

**Simulation details of comparison analysis**

Simulated data were created using SimAdmix. For all runs, all 39,905 SNPs on chromosome 22 from HapMap 3 release 2 build 36 were used with a track length of 40 centimorgans, with 50 samples created. For two-way admixture, simulated African-American data were created using 120 samples of CEU (Western European) and 120 samples of YRI (Northwestern African) haplotypes with proportions of .20 and .80, respectively, also from the same HapMap source as the SNP data. For three-way admixture, samples of simulated Latino data were created with the same CEU and YRI data, and 180 samples of Han Chinese (CHB) haplotypes in place of Native American (as the amount of reference data for the latter is scarce) with respective proportions of .60, 30, and .10. ELAI and LAMP-LD’s outputs were converted to unphased genotypes to ensure consistency across comparisons between the remaining software, as they can output phased haplotypes while LAMP and HAPMIX’s outputs are unphased.

For real data analysis, we did runs on 758 samples of African-Americans on chromosome 21, with 87,569 SNPs used in analysis. The reference haplotypes were the same as used in the simulated datasets. In order to calculate the correlations of local ancestry inference results, the subset of each software’s SNPs that were included in analysis (after filtering from LAIT) had to be used – 28,625 SNPs were used for these calculations.

All inference software were run with default and/or recommended parameters from each of their respective manuals. All statistics were calculated and recorded using in-house Perl, R, and Bash scripts. Trials were run using an Intel Xeon E5-2695 v2 at 2.40GHz (none of the software supports multithreading).
